# Antimicrobial Resistance Phenotype of *Staphylococcus aureus* and *Escherichia coli* Isolates Obtained from Meat in the Formal and Informal Sectors in South Africa

**DOI:** 10.1155/2020/3979482

**Published:** 2020-09-19

**Authors:** Ishmael Festus Jaja, Chinwe-Juliana Iwu Jaja, Nnamdi Vincent Chigor, Madubuike Umunna Anyanwu, Ezealisiji Kenneth Maduabuchi, James Wabwire Oguttu, Ezekiel Green

**Affiliations:** ^1^Department of Livestock and Pasture Science, University of Fort Hare, Alice 5700, South Africa; ^2^Department of Agriculture and Animal Health, University of South Africa, Roodepoort Johannesburg 1710, South Africa; ^3^Department of Nursing and Midwifery, Faculty of Medicine and Health Sciences, Stellenbosch University, Cape Town 7505, South Africa; ^4^Department of Microbiology, Faculty of Science, University of Nigeria, Nsukka, Nigeria; ^5^Microbiology Unit, Department of Veterinary Pathology and Microbiology, University of Nigeria, Nsukka, Nigeria; ^6^Department of Pharmaceutical Chemistry, Faculty of Pharmaceutical Sciences, University of Port Harcourt, Port Harcourt, Nigeria; ^7^Department of Biotechnology and Food Science, Faculty of Science, University of Johannesburg, Doornfontein 2028, South Africa

## Abstract

**Background:**

Foodborne diseases (FBD) caused by resistant pathogens are a global public health problem. One main driver of the increasing FBD incidence is the transfer of pathogenic organisms from animal guts to carcasses during processing and subsequent transfer from meat products to consumers.

**Methods:**

In this study, meat samples from abattoirs in the formal meat sector (FMS) (*n* = 140) and slaughter points in the informal meat sector (IMS) (*n* = 104) were collected for microbial detection and phenotypic AMR determination using polymerase chain reaction.

**Results:**

The antibiogram of *Staphylococcus aureus* isolates revealed that resistance to clindamycin (74.3%) and ampicillin (59.5%) was highest in the FMS, while resistance to penicillin (83.8%) and tetracycline (82.1%) was highest in the IMS. *Escherichia coli* isolates show significant resistance to chloramphenicol (90.7%) and tetracycline (82.3%) in the FMS. Likewise, resistance to tetracycline (92.3%) and sulfamethoxazole/trimethoprim (87.5%) was highest in the IMS. The multiple antibiotic resistance index (MARI) for *S*. *aureus* and *E*. *coli* ranged from 0.3 to 0.8 and 0.2 to 0.5, respectively.

**Conclusion:**

This study suggests high-level contamination of meat with resistant pathogens and highlights the public health consequences associated with consuming such unhygienic products.

## 1. Introduction

Meat is an essential source of animal protein widely consumed in many parts of the world. In terms of livestock agriculture, statistics show that there about 1.1 million pigs, 7 million goats, 24.6 million sheep, 1.4 million dairy cattle, and 13.6 million beef cattle as well as 1.6 million ostriches, 31.8 million layers, and 113 million broilers in South Africa [1]. South Africa meat consumption per capita per year is said to be 41 kg and is second only to Ghana in Africa [2]. In the Southern African region, meat consumption is four times higher than any other region in Africa and South Africa plays a major role regarding livestock production and meat supply in the continent [1, 3].

Even though meat plays a crucial role in human nutrition, a significant proportion of foodborne diseases have been linked to its consumption. Epidemiological data suggest an escalating incidence of foodborne diseases. A good number of these diseases occur due to poor animal husbandry systems and failure to maintain proper hygiene during food processing [2, 4]. Poor hygiene management and other faulty abattoir processes such as improper evisceration increase the chances of cross-contamination of gut pathogens (*Escherichia coli*, *Salmonella* spp., *Campylobacter* spp., *Staphylococcus aureus*, and enteric bacteria) to meat [5].

*Escherichia coli* is part of the normal flora of the gastrointestinal tract of humans and animals. It becomes pathogenic to the immunocompromised person (children, pregnant mothers, and people with a chronic debilitating illness such as diabetes) through contaminated water and food [6, 7]. Many *E*. *coli* strains have emerged as leading zoonotic foodborne pathogens. Diarrheagenic pathotypes frequently implicated foodborne for diseases include enterotoxigenic *E*. *coli* (ETEC), enteropathogenic *E*. *coli* (EPEC), Shiga toxin-producing *E*. *coli* (STEC), enteroaggregative *E*. *coli* (EAEC), enterohemorrhagic, diffusely adherent *E*. *coli* (DAEC), and *E*. *coli* (EHEC); a subclass of enteroinvasive *E*. *coli* (EIEC), neonatal meningitis *E*. *coli* (NMEC), *E*. *coli*, and uropathogenic *E*. *coli* (UPEC)[8–10]. Due to their ability to cause numerous foodborne disease outbreaks in humans, they have become a significant public health threat [7, 11–13].

*Staphylococcus aureus* is among the leading causes of foodborne diseases in humans. It is a Gram-positive, nonspore forming, nonmotile, catalase-positive coccus which is ubiquitous in humans and the environment [14]. *S*. *aureus* is found commonly on the skin, hair, noses, and respiratory tract of humans and animals. It multiplies rapidly at room temperature producing toxins which cause illnesses when it enters the body. The main route of transmission of *S*. *aureus* is through a cut, infected wound, and ingestion of contaminated food [15].

*Staphylococcus aureus* is commonly associated with intoxications due to its ability to produce a variety of potent staphylococcal enterotoxins (SEs) [7]. The SEs are resistant to inactivation by GIT proteases such as pepsin and display strong thermoresistance, an essential property of SEs for food safety considerations and a potential problem for public health [16]. *Staphylococcus aureus* produces three types of hemolysins, known as alpha, beta, and delta toxins. The beta-hemolysin gene encodes the beta toxins that inhibit the ciliary movement of human lungs and corneas [17]. Due to its transient nature, many staphylococcal food poisonings (SFP) go unreported; this is in addition to the fact that the symptoms of SFP are similar to those of food poisoning caused by *Bacillus cereus* [18].

In humans, gastroenteritis attributable to staphyloenterotoxicosis or staphyloenterotoxemia can occur within 1 to 7 hours after consumption of contaminated food [19]. Dehydration due to frequent diarrhea and vomiting; infections of the skin; and soft tissue, joint, bone, respiratory, and endovascular disorders are other common clinical pictures in infected humans. Furthermore, diseases such as pneumonia, meningitis, osteomyelitis, endocarditis, and toxic shock syndrome are commonly associated with staphylococcal infection [15]. Further compounding the challenges posed by staphylococcus infection is the increasing spate of methicillin-resistant *S*. *aureus* (MRSA), which have been reported in pork, chicken, beef, and other meat in many countries [20–23].

In many developing countries, the incidence of foodborne diseases (FBD) is often associated with resistant bacteria [6, 12, 24]. Food-associated microbes harboring transferable antibiotic resistance genes are of significant public health concern. This is because they can cause FBD and also act as a reservoir for spreading antibiotic resistance genes to enteric and commensal bacteria by horizontal gene transfer of mobile genetic elements [25]. The problem of antibiotic resistance could even be more prominent in South Africa given that farmers under the Stock Remedies Act (Act 36 of 1947) could buy and use the veterinary drug without a prescription [26–28]. Hence, this study is aimed at determining the antimicrobial resistance profile of *Staphylococcus aureus* and *Escherichia coli* isolates from raw meat, slaughtered carcasses in the informal and informal meat sectors in the Eastern Cape Province of South Africa.

## 2. Material and Method

### 2.1. Ethical Approval

Approval for this research was obtained from the University of Fort Hare Research and Ethics Committee (UREC). The certificate of approval was issued with reference number MUC351SJAJ01.

### 2.2. Study Area

The study was conducted at two high-throughput abattoirs (HT1 and HT2). The East London abattoir (HT1) is situated at 32.97°S and 27.87°E in the Buffalo City Metropolitan Municipality, while the Queenstown abattoir (HT2) is located 31°54′S and 26°53′E in the Chris Hani District of the Eastern Cape Province [5, 29]. The informal slaughter point was Alice (32.47°S and 26.50°E), King William's Town (32°53′S and 27°24′E), Queenstown (31°54′S and 26°53′E), and East London (32.97°S and 27.87°). The places receive approximately 480-850 mm of rainfall per year most of which is during the summer months and are situated about 586-2371 meters above sea level. The ambient temperatures in the Eastern Cape during the period of study ranged from 18°C to 39°C with mean temperatures of 20.5°C. The vegetation in this area is composed of bushveld with *Acacia karroo*, *Themeda triandra*, and *Digitaria eriantha*, grasslands, and forests. The predominant farming system is extensive with some commercial farms using a semi-intensive system of management [5, 29].

### 2.3. Sample Collection and Sampling Design

Swab samples from carcasses of slaughtered animals were collected from November 2016 to October 2017 at the formal and informal meat sectors. The formal refers to livestock producers who are registered with the Provincial Departments of Agriculture and whose activities are governed by relevant acts of the national parliament. The informal livestock producers, on the other hand, are a subset of unincorporated enterprise, with less than a specialized size in terms of the number of persons employed, and may or may not be registered under specific forms of national legislation [30]. Enterprises in the informal sector do not pay tax and/or obey employment regulations and are rarely monitored for health and safety standards [31, 32]. In this regard, the abattoir represents the formal meat sector, while the backyard and unapproved slaughter points were included as the informal meat sector.

Carcasses were sampled according to the United States Department of Agriculture Food Safety and Inspection Service (FSIS) protocol on livestock carcass examination. This protocol has been outlined elsewhere [33]. For HT1 and HT2, a simple random sampling technique was adopted for the survey; this sampling method allowed for the convenient swabbing of animal carcasses. In the informal meat sector, a snowball technique was used to identify informal slaughter points for sample collection and carcasses were purposively sampled based on the available number of slaughtered animals. A total of 244 carcasses were sampled from two high-throughput abattoirs represented as HT1 (168 cattle) and HT2 (36 sheep and 40 pigs). In the informal meat sector, a total of 136 swab samples (52 cattle and 84 sheep) were collected. Samples were aseptically collected using cotton throat sponges (CTS) hydrated with 10 ml of buffered peptone water (BPW) (Inqaba® Laboratories, South Africa). All the carcasses were sampled using the same swabbing technique at the end of the slaughter line after dressing but before chilling. The technique entails a horizontally and vertically directed swabbing across the sampling site (neck, brisket, flack, and ramp) on a total of 100 cm^2^ quadrant forming a pooled sample for each carcass [34]. Each of the areas on the 4 quadrants of the carcass was firmly swabbed repetitively and abrasively ensuring that most if not all bacteria on the meat surface were removed onto the CTS. Samples were labeled and carefully packed in a cooler box containing ice packs and transported to the laboratory on the same day for bacterial analysis.

### 2.4. Isolation of *Staphylococcus aureus* and *Escherichia coli*

Each polled sample was inoculated into tryptone soy broth (TSB) (Merck, SA) and incubated for 24 h at 37°C. A loop of liquid was removed from the cultures and streaked onto mannitol salt agar (MSA) (Biolab, Midrand, South Africa) plates for *Staphylococcus aureus* isolation and eosin methylene blue agar (EMB) (Oxoid, Basingstoke, UK) for *Escherichia coli* isolation. *S*. *aureus* was presumed to be positive if yellow or off-white colonies were found on MSA, indicating mannitol fermentation (i.e., presumptive coagulase-positive staphylococci). Salt tolerance and mannitol fermentation properties of *S*. *aureus* produced the typical yellow colonies because of the change in pH [35]. Further confirmation was done by Gram staining and standard biochemical assays such as catalase, oxidase, and coagulase testing [36]. After incubation, colonies with a distinct green metallic sheen on EMB were regarded as *E*. *coli* [35]. All identified presumptive colonies were kept in glycerol stock and then stored at −80°C for further analyses.

### 2.5. DNA Extraction

Bacterial deoxyribonucleic acid was extracted from presumptive isolates using the boiling method as described elsewhere [6, 37]. Briefly, the bacteria stored in glycerol stocks were first resuscitated by inoculation into TSB (Merck, SA) and incubated at 37°C for 24 h. Finally, a loop of liquid was removed from TSB and streaked onto nutrient agar (Merck, SA) and incubated at 37°C for 24 h. DNA extraction was performed using a boiling method. The method entails selecting 3–5 colonies using a sterile wire loop into sterile DNAse/RNAse-free Eppendorf tubes (Biologix, USA) containing 200 *μ*l nuclease-free water (Thermo Scientific, USA). Each suspension was vortexed using a minishaker (Digisystem Laboratory Instruments Inc., Taiwan), and the cells were lysed using a Dri-Block DB-2A (Techne, South Africa) for 15 min at 100°C. The Eppendorf tubes were then incubated in a heat block at 100°C for 15 min and then kept on ice before the final centrifugation at13,000 rpm for 5 min for removal of cell debris. The supernatant was collected into a sterile Eppendorf tube and preserved at −20°C until further tests.

### 2.6. Molecular Identification Using Polymerase Chain Reaction

**Figure 1:**
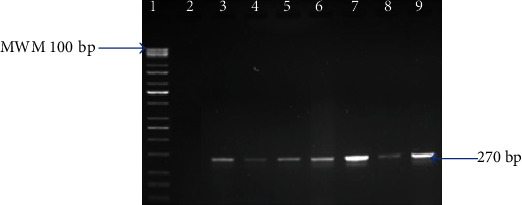
Gel image of amplified PCR products from study isolates with primers designed for the *Nuc* gene. Lane 1 is the MWM (100 bp); lane 2 is the negative control (PCR mix without DNA) with lane 3 as the positive control (ATCC® 25923), while lanes 4 to 9 are *Nuc* (270 bp) gene amplified from *S*. *aureus* isolates.

**Figure 2:**
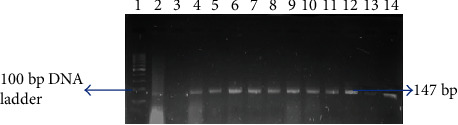
Gel image of amplified PCR products from study isolates with primers designed for the *uidA* gene. Lane 1 is the 100 bp ladder; lane 2 is the negative control (PCR mix without DNA) with lane 3 as the positive control (ATCC® 25922), while lanes 4 to 14 are positive *E*. *coli* isolates with amplified gene (147 bp).

Molecular confirmation of presumptive S. aureus and E. coli isolates was done by PCR using a primer pair to target the thermonuclease (Nuc) gene for S. aureus [14, 38] and uidA gene for E. coli [39, 40] (Figures 1 and 2). Quality control strains S. aureus ATCC 25923 and E. coli ATCC 25922 served as positive controls. Negative controls were used in all reactions containing the reaction mixture except the DNA template, which was replaced by nuclease-free water. The reaction mixture for running PCR contained 12.5 *μ*l of 2x DreamTaq PCR master mixes (Thermo Scientific, SA), 5.5 *μ*l nuclease-free water, 1 *μ*l of both the primers, and 5.0 *μ*l of the DNA template. PCR assay was carried out in a 25 *μ*l reaction volume. The thermocycling program for PCR can be found in Table 1. The amplified products were visualized by standard gel electrophoresis using 5 *μ*l of the amplified product on 2% agarose gels immersed in 0.5x TBE buffer. The TBE buffer contained 0.1 M Tris, 0.1 M boric acid, and 0.002 M NaEDTA. Agarose gels were stained using 1 mg/ml ethidium bromide and photographed under UV light with a transilluminator (Alliance 4.7).

### 2.7. Antimicrobial Susceptibility Testing

Antibiotic susceptibility testing was performed by the Kirby-Bauer disc diffusion test method, following the guidelines of the Clinical and Laboratory Standards Institute [41]. An inoculum of each pure bacterial isolate was emulsified in 5 ml of sterile normal saline, and the density was adjusted to 0.5 McFarland standards. A sterile cotton swab was dipped into the standardized suspension of bacterial cultures and used to inoculate Mueller Hinton agar (MHA) plates, and the plates were allowed to dry. Antibiotic discs with the following drug contents ampicillin (10 *μ*g), erythromycin (15 *μ*g), rifampicin (5 *μ*g), clindamycin (2 *μ*g), ciprofloxacin (5 *μ*g), penicillin (10 *μ*g), tetracycline (30 *μ*g), chloramphenicol (30 *μ*g), gentamycin (10 *μ*g), trimethoprim-sulfamethoxazole (25 *μ*g), amikacin (30 *μ*g), and ofloxacin (5 *μ*g) were placed onto Mueller Hinton agar (MHA) plates using a disc diffuser (DMM063, Thermo Fisher Scientific, South Africa). The plates were incubated at 37°C for 24 hours. The zone diameter was measured using a ruler, and results were interpreted according to Clinical and Laboratory Standards Institute (CLSI) guidelines [41].

### 2.8. Statistical Analysis

All data analysis was performed using Microsoft® Excel (2007) mathematical functions and Statistical Package for the Social Sciences (SPSS) version 22 (SPSS Inc., Chicago, IL). Exploratory data analysis was used to validate the data and calculate crude associations by using2 × 2cross-tabulation tables in which descriptive statistics and summary measures were calculated. Multiple antibiotic resistance phenotypes (MARPs) for *S*. *aureus* isolates from the formal and informal meat sectors were then generated for isolates that were resistant to five or more antimicrobials [39]. The frequencies, percentages, and number of antimicrobials to which the isolates were resistant and resistance patterns were obtained from the antimicrobial susceptibility testing (AST). The multiple antibiotic resistance indexes (MARI) for bacterial isolates from both meat sectors were mathematically calculated using MAR_index_ = *a*/*b*, where *a* stands for the number of antibiotics to which the isolate was resistant and “*b*” represents the total number of antibiotics used for antimicrobial susceptibility testing [42].

## 3. Results

### 3.1. Prevalence and Antibiogram of *Staphylococcus aureus* and *Escherichia coli* in the Formal and Informal Meat Sectors

**Figure 3:**
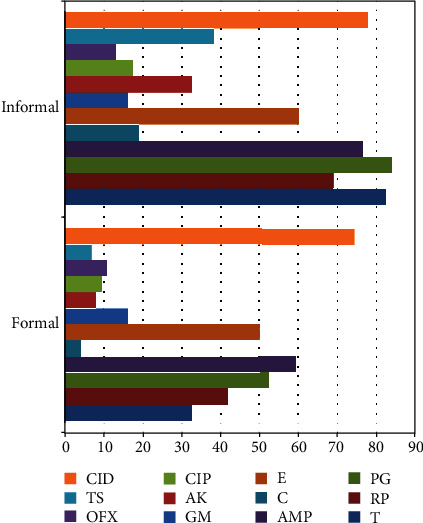
Percentage phenotypic resistance profile of *S*. *aureus* isolates from the formal and informal meat sectors. T: tetracycline; RP: rifampicin; PG: penicillin; AMP: ampicillin; C: chloramphenicol; E: erythromycin; GM: gentamycin; AK: amikacin; CIP: ciprofloxacin; OFX: ofloxacin; TS: sulfamethoxazole/trimethoprim; CID: clindamycin.

**Figure 4:**
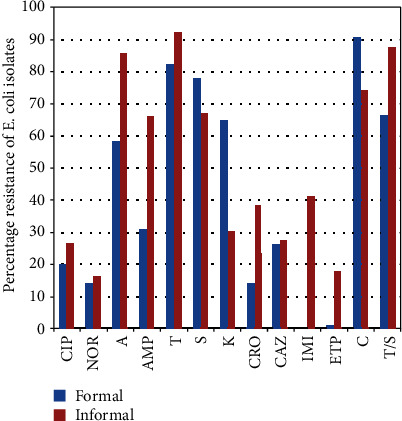
Percentage phenotypic resistance profile of *E*. *coli* isolates from the formal and informal meat sectors. T: tetracycline; PG: penicillin; AMP: ampicillin; C: chloramphenicol; CIP: ciprofloxacin; TS: sulfamethoxazole/trimethoprim; S: streptomycin; CRO: ceftriaxone; CAZ: ceftazidime; IMI: imipenem; ETP: ertapenem; NOR: norfloxacin; A: amoxicillin.

The prevalence of molecularly confirmed *Staphylococcus aureus* in the formal and informal meat sectors was 30.3% (74/244) and 50% (68/244), respectively (Table 2). The molecularly confirmed *E*. *coli* in the formal and informal meat sectors was 57.4% (140/244) and 76.5% (104/244) (Table 2). For *S*. *aureus* isolates, resistance to clindamycin 74.3% (55/74) was highest in the FMS (Figure 3), followed by ampicillin 59.5% (44/74), penicillin 52.7% (32/74), and erythromycin 50% (37.74), whereas resistance to penicillin 83.8% (67/68) was highest for the IMS, followed by tetracycline 82.4% (56/68), clindamycin 77.9% (52/68), ampicillin 76.5% (52/68), and rifampicin 69.1% (47/68) (Table 3). Two isolates each showed multiple drug resistance to 9 and 10 antibiotics, respectively. The multiple antibiotic resistance indexes (MARI) for the formal and informal meat sectors ranged from 0.3 to 0.8 (Table 4). For *E*. *coli* isolates, resistance to chloramphenicol 90.7% (127/140), tetracycline 82.1% (115/140), streptomycin 77.9% (109/140), sulfamethoxazole/trimethoprim 66.4% (93/140), kanamycin 65% (91/140), and amoxicillin 58.6% (82/140) was highest in the formal meat sector (Figure 4). All isolates were susceptible to imipenem (Table 5). *E*. *coli* isolates obtained from the informal meat sector were mostly resistant to tetracycline 92.3% (96/104), sulfamethoxazole/trimethoprim 87.5% (91/104), amoxicillin 85.6% (89/104), chloramphenicol 74% (77/104), streptomycin 67.3% (70/104), and ampicillin 66.3% (69/104) (Table 5). Four and 14 *E*. *coli* isolates were resistant to 10 antibiotics, and the MARI for these isolates was 0.5 (Table 6).

## 4. Discussion

Meat consumers in low- and middle-income countries obtain meat from the informal outlets because the meat is cheap, and the market is often situated close to rural communities [31, 43]. However, in the absence of proper meat safety and hygiene management systems, the chemical constituent of meat enhances microbial growth to unacceptable levels. Hence, microbially compromised meat poses the risk of foodborne disease (FBD) transmission to consumers. The prevalence of FBD is a growing public health problem especially in low- and middle-income countries where food safety systems are poorly implemented [23, 44–46].

Foodborne pathogens such as *E*. *coli*, *Salmonella*, and *Campylobacter* are excreted from the gastrointestinal tract of food-producing animals, and cross-contamination is often as a result of poor slaughter technique and hygiene standard at abattoirs [2, 11, 47–49]. The occurrence of food-related disease is further compounded by the development of antimicrobial resistance (AMR) by bacteria, which limits the efficiency of antibiotic therapeusis. The present study investigated the level of microbial contamination of slaughtered carcasses in the formal and informal meat sectors.

The present study also investigated the antimicrobial resistance (AMR) profile of *S*. *aureus* and *E*. *coli* isolates obtained from the formal and informal meat sectors (Figures 3 and 4). *Staphylococcus aureus* isolates from the formal meat sector were mostly resistant to rifampicin (41.9%), penicillin (52.7%), ampicillin (59.5%), erythromycin (50%), and clindamycin (74.3%) (Figure 3). In the informal meat sector, *S*. *aureus* isolates were mainly resistant to tetracycline (82.4%), penicillin (83.8%), and ampicillin (76.5%) which demonstrates the growing problem of AMR in bacteria from food-producing animals. The resistance to important antibiotics such as rifampicin (69.1%), erythromycin (60.3%), and clindamycin (77.9%) is even more worrisome. One study of poultry meat in South Africa found high resistance to tetracycline in all *S*. *aureus* [50]. In another study, the resistance to clindamycin was 11.8% for beef cuts and 21.7% for pork. The same study found the resistance to penicillin to be 63.2% for beef cuts and 88.7% for pork [48].

Approximately 20% and 30% of humans are regarded as persistent and intermittent carriers of *S*. *aureus* in the nostrils, respectively. Thus, *S*. *aureus* asymptomatically lives on the skin and nostrils of humans and animals [23, 35]. Cross-contamination from the animal and human skin to the meat during slaughter and processing is inevitable in a situation where the standard hygiene protocol is not strictly implemented. A practical example of the persistence of *S aureus* in human hands and nares was demonstrated in one Brazilian study, where methicillin-resistant *Staphylococcus aureus* (MRSA) was detected in 28.6% of samples collected from the hands and nares of food handlers in a public hospital. The finding in the Brazilian study reinforces the need for strict sanitary protocols at meat handling points. It further supports our hypotheses that some of the isolates in the present study were a result of cross-contamination from slaughter personnel.

Maintaining hygiene and safety in the informal market is challenging to achieve because many traders are not educated or poorly resourced to implement the standard hygiene protocol. Many of the informal traders wash meat repeatedly in the same water without change, using the same knife the entire day without cleaning or washing in hot water. Temperature violation was common, and meat is usually not efficiently protected from flies and dust that may harbor meatborne pathogens. Poor hygienic behaviors observed in the present study are consistent with previous studies [43, 51]. In these kinds of condition, it is easy for pathogens to be transferred from meat handlers, knives, flies, dust, and tables [35, 52–54]. *Staphylococcus aureus* as a potential pathogen may adversely affect animal and human health by causing abscesses, endocarditis, severe necrotic lesions, and bacteremia [36]. Bacteria harboring resistant determinant and virulence factors could quickly disseminate these factors through mobile genetic element coding for the transfer of resistance horizontally between various bacteria.

Foodborne illnesses caused by *S*. *aureus* are a result of the ingestion of food contaminated with staphylococcal toxins. Staphylococcal enterotoxins are 23 to 29 kDa single-chain proteins that also possess immunomodulation properties [55] and are mostly carried on mobile genetic elements that aid their horizontal transfer between bacterial populations [19, 56]. The implication of these resistance proportions can be seen in the high MAR index of 0.4-0.8 for isolates from the formal and informal meat sectors. The high levels of *S*. *aureus* and *E*. *coli* recovered in this study may pose a public health hazard due to the potential pathogenicity and/or toxigenicity of various strains of these bacteria.

Even though food safety systems and standardization are widely applied in the formal meat sector, unlike the informal sector where there is no regulation governing the safety of meat [57, 58], the microbial quality of meat in the formal meat sector hardly reflects these standards. This is especially true in this instance, given that in this study, 91 and 98 *E*. *coli* isolates were resistant to either three or more antibiotics (Table 6). Antimicrobial resistance to chloramphenicol (90.7%) and tetracycline (92.3%) was highest in the formal and informal meat sectors, respectively. Although chloramphenicol use in veterinary medicine has been restricted globally [26, 59], its detection in high proportion suggests that carcasses from the study sites were heavily contaminated with pathogens of human or environmental origin. On the other hand, the high resistance to tetracycline is unsurprising given that it is a common over-the-counter (OTC) medication for the treatment of bacteria and tick-borne diseases (TBDs) in South Africa [37, 60]. In many instances, farmers misapply tetracycline to treat unrelated diseases. Such imprudent use of antibiotics exerts selective pressure sustaining the emergence of resistant bacterial strains.

Streptomycin (77.9% and 67.3%), sulfamethoxazole/trimethoprim (66.4% and 87.5%), and amoxicillin (58.6% and 85.6%) were the other antibiotics with high resistant proportion in the formal and informal meat sectors, respectively. The use of antibiotics for prophylaxis, metaphylaxis, and growth promotion in livestock farms is the primary suspect in selecting antibiotic resistance. Antimicrobial agents such as sulfonamides (95.4%); macrolides, lincosamides, and pleuromutilins (61.6%); tetracyclines (14%); quinoxalines (8.2%); lonophores (6.7%); and penicillins (1.8%) have been reportedly sold as in-feed and water antimicrobials medication [60].

The scale of resistant pathogens obtained in this study remains worrisome. Even more worrisome is the growing resistance to third-generation antimicrobial agents such as ceftriaxone, ceftazidime, imipenem, and ertapenem (Table 5). The main driver for cephalosporin remains unclear since it is not commonly used in animal medicine. Hence, we suspect that the extensive use of cephalosporins and carbapenems in clinical practice may play a role in the current resistant profile. Also, antimicrobial resistance to third-generation cephalosporins is frequently related to the production of extended-spectrum *β*-lactamase (ESBL) enzymes. Aside from ESBLs, antimicrobial resistance to extended-spectrum cephalosporinases (ESCs) in *E*. *coli* has been associated with plasmid-mediated Ambler class C cephamycinases [61].

The fact that pathogens from animals spread to food products during slaughter and processing has been extensively published [33, 35, 62]. Bacteria with resistance capability can also be transferred from animals and humans during slaughter and processing [37, 63]. More important is the misappropriation of antimicrobial agents by communal farmers who are the primary suppliers of meat in the informal market [32, 57, 64, 65]. Poorly resourced farmers lack adequate farm infrastructures necessary for modern livestock production [66]. Infrastructures such as crush pans and digital weighing scales are needed to weigh animals to aid proper dosage of medicine for animal prophylaxis and therapy. Thus, during antibiotic administration, animals could be given a suboptimal dose or overdose of antimicrobial agents. Moreover, the acute shortage of veterinary skilled labor further compounds the problem of AMR as veterinarians are responsible for primary animal health care [67]. Hence, farmers often resort to self-medicating their animals.

The misapplication of antibiotics selects for AMR and the transfer of resistance determinants to other bacteria population. This could fuel the spread of antibiotic-resistant bacteria (ARB), imposing a heavy burden on the health of humans and animals. A high circulating ARB further increases the burden of disease in the community and length of hospitalization of sick human patients.

## 5. Conclusion

This study demonstrated that multiple antibiotic resistance phenotypes (MARPs) were present in *S*. *aureus* and *E*. *coli* isolates obtained from meat in the formal and informal meat sectors. The overall resistance rate was high such as clindamycin, ampicillin, rifampicin, streptomycin, and amoxicillin. The bacterial isolates showed a high MARI of 0.2 to 0.5; however, the informal sector presented a higher number of MARPs than the formal sector, demonstrating a highly compromised hygiene environment for the processing of meat. Even though all samples from the formal meat sector were collected after carcass washing, the prevalence of *E*. *coli* in meat is disturbing, given that these are export abattoirs with established hygiene management systems. Because complete eradication of bacteria may not be possible, transmission control seems to be an appropriate goal. Some control methods are widely recognized as effective. Of these methods, the first and most effective method is to avoid transmission through hand contamination from slaughter personnel to animal carcass.

There is also an urgent need for policy formulations on the prudent use of antimicrobials in both human and veterinary medicine. Farmers in the formal and informal meat sectors need to be adequately educated about antibiotic stewardship and implication of the persistent indiscriminate use of antimicrobial agents. Likewise, butchers at the abattoir, and other slaughter points in the informal meat sector, should be educated on good slaughter and hygiene techniques. There is still a big gap in understanding the genetic background of antibiotic resistance and virulence of bacteria from food sources. Further study on the genotypic characterization of resistance in bacteria and its pathogenicity is suggested. Furthermore, whole-genome sequencing of isolated bacteria will aid the tracing of the source of contamination.

## Figures and Tables

**Table 1:** Primers used in PCR detection of *S*. *aureus* and *E*. *coli*.

Gene			Reference
Nuc	Primer sequence 5′-3′	F: GCGATTGATGGTGATACGGTT	[35]
		R: AGCCAAGCCTTGAACGAACTAAAGC	
	Product size (bp)	270	
	PCR conditions	Initial denaturation at 95°C for 5 min was followed by 37 cycles of amplification (denaturation at 95°C for 30 s, annealing at 55°C for 30 s, and extension at 72°C for 60 s) and ending with a final extension at 72°C for 10 min	

uidA	Primer sequence 5′-3′	F: AAAACGGCAAGAAAAAGCAG	[35]
		R: ACGCGTGGTTAACAGTCTTGCG	
	Product size (bp)	147	
	PCR conditions	Initial denaturation at 94°C for 2 min followed by 25 cycles of denaturation at 94°C for 1 min, annealing at 58°C for 1 min, and extension at 72°C for 1 min and ended with a final extension at 72°C for 2 min. Holding was at 4°C	

**Table 2:** Percentage isolation of *S*. *aureus* and *E*. *coli* in the formal and informal meat sectors.

Meat sector	Abattoirs/slaughter points	Animal	No. of carcasses sampled	*S*. *aureus*		*E*. *coli*	
				Presumptive isolates (%)	Confirmed with PCR (%)	Presumptive isolates (%)	Confirmed with PCR (%)
Formal	HT1	Cattle	168	143 (58.6)	51 (20.9)	109 (44.7)	104 (42.6)
Formal	HT2	Sheep	36	23 (9.4)	7 (2.9)	27 (11.1)	24 (9.8)
		Pig	40	31 (12.7)	16 (6.6)	21 (8.6)	12 (4.9)
Total			244	197 (80.7)	74 (30.3)	157 (64.3)	140 (57.4)
Informal	Alice	Cattle	16	16 (11.8)	10 (7.4)	16 (11.8)	14 (10.3)
		Sheep	32	31 (22.8)	9 (6.6)	21 (15.4)	20 (14.7)
Informal	East London	Cattle	20	20 (14.7)	11 (8.1)	16 (11.8)	16 (11.8)
Informal	King William's Town	Cattle	16	15 (11)	9 (6.6)	11 (8.1)	11 (8.1)
Informal	Queenstown	Sheep	52	46 (33.8)	29 (21.3)	44 (32.4)	43 (31.6)
Total			136	128 (94.1)	68 (50)	108 (79.4)	104 (76.5)

**Table 3:** Antibiotic susceptibility pattern of *Staphylococcus aureus* isolates in the formal (*n* = 74) and informal (*n* = 68) meat sectors.

Antibiotic class	Antimicrobial agents	Code	Potency (*μ*g)	Formal		Informal	
				S (%)	R (%)	S (%)	R (%)
Tetracycline	Tetracycline	T	30	50 (67.6)	24 (32.4)	12 (17.6)	56 (82.4)
Ansamycins	Rifampicin	RP	5	43 (58.1)	31 (41.9)	21 (30.9)	47 (69.1)
Penicillin	Penicillin	PG	10	35 (47.3)	39 (52.7)	11 (16.2)	57 (83.8)
	Ampicillin	AMP	10	30 (40.50)	44 (59.5)	16 (23.5)	52 (76.5)
Phenicols	Chloramphenicol	C	10	71 (95.9)	3 (4.1)	55 (80.9)	13 (19.1)
Macrolides	Erythromycin	E	15	37 (50)	37 (50)	27 (39.7)	41 (60.3)
Aminoglycosides	Gentamycin	GM	10	62 (83.8)	12 (16.2)	57 (83.8)	11 (16.2)
	Amikacin	AK	30	68 (91.9)	6 (8.1)	46 (67.6)	22 (32.4)
Quinolones	Ciprofloxacin	CIP	5	67 (90.5)	7 (9.5)	56 (82.4)	12 (17.6)
	Ofloxacin	OFX	5	66 (89.2)	8 (10.8)	59 (86.8)	9 (13.2)
Foliate pathway inhibitor	Sulfamethoxazole/trimethoprim	TS	25	69 (93.2)	5 (6.8)	42 (61.8)	26 (38.2)
Lincosamides	Clindamycin	CID	2	19 (25.7)	55 (74.3)	15 (22.1)	53 (77.9)

S: susceptible; R: resistance.

**Table 4:** Multiple antibiotic resistance patterns (MARPs) and MARI of *Staphylococcus* spp. from the formal and informal meat sectors.

S/no.	Isolate code	Resistance pattern	No. of antibiotics	MARI	S/no.	Isolate code	Resistance pattern	No. of antibiotics	MARI
1	C29C^INMS^	RP-CD-AP-E-PG	5	0.4	20	C3A	RP-CD-AP-E-PG	5	0.4
2	C18A^INMS^	CD-GM-CIP-AK	4	0.3	21	C1A^INMS^	RP-CD-AP-E-T-PG	6	0.5
3	C29B	RP-CD-AP-E-T-PG	6	0.5	22	C24C	RP-C-CD-AP-E-T-GM-TS-PG-AK	10	0.8
4	C3D^INMS^	AP-E-GM-OFX	4	0.3	23	P9C	CD-AP-E-T	4	0.3
5	C22A^INMS^	RP-CD-AP-E-T-GM-PG	7	0.6	24	C17C	RP-CD-AP-E-PG	5	0.4
6	C20A	RP-CD-AP-E-T-PG	6	0.5	25	17SD^INMS^	RP-CD-AP-E-T-GM-PG	7	0.6
7	C22C	RP-CD-AP-E-GM-CIP-OFX-PG-AK	9	0.8	26	C28B	RP-CD-E-PG	4	0.3
8	C5A^INMS^	CD-AP-E-T-PG	5	0.4	27	C28D	RP-CD-AP-E-OFX-PG	6	0.5
9	C28C	RP-CD-AP-E-PG	5	0.4	28	C7D^INMS^	CD-AP-GM-TS-OFX-PG	6	0.5
10	17SA	CD-AP-E-T-GM-PG	6	0.5	29	C3D^INMS^	CD-AP-E-T	4	0.3
11	C16A	RP-CD-AP-E-PG	5	0.4	30	C30D	RP-CD-AP-E-T-PG	6	0.5
12	C3C^INMS^	RP-CD-AP-T-GM-PG-AK	7	0.6	31	C24A	RP-CD-AP-E-PG	5	0.4
13	C26D	RP-CD-AP-E-PG	5	0.4	32	C27C^INMS^	RP-CD-AP-E-T-PG	6	0.5
14	14SD	RP-CD-AP-E-GM-CIP-OFX-PG-AK	9	0.8	33	C20A^INMS^	C-CD-AP-E-T-GM-TS-CIP-OFX-PG	10	0.8
15	DH5	RP-CD-E-T	4	0.3	34	C4C^INMS^	RP-CD-AP-E	4	0.3
16	12SC^INMS^	RP-CD-AP-E-T-PG	6	0.5	35	C30A	RP-CD-AP-E-PG	5	0.4
17	26SA	RP-CD-AP-E-PG	5	0.4	36	SM11	RP-CD-AP-E-T-GM	6	0.5
18	C18A^INMS^	RP-CD-AP-E-TS-CIP-OFX	7	0.6	37	P9B	RP-CD-AP-PG	4	0.3
19	C27D	RP-CD-AP-PG	4	0.3	38	P5A	RP-CD-AP-T-PG	5	0.4

Isolates with superscript ^INMS^ were from the informal meat sector; those without superscript were from the formal sector.

**Table 5:** Percentage antibiotic susceptibility of *E*. *coli* isolates in the formal (*n* = 140) and informal (*n* = 104) meat sectors.

Antimicrobial class	Antimicrobials	Disc code	Potency (*μ*g)	Meat sector			
				Formal		Informal	
				R (%)	S (%)	R (%)	S (%)
Quinolones	Ciprofloxacin	CIP	5	28 (20)	112 (80)	28 (26.9)	76 (73.1)
	Norfloxacin	NOR	10	20 (14.3)	120 (85.7)	17 (16.3)	87 (83.7)
Beta-lactams	Amoxicillin	A	25	82 (58.6)	58 (41.4)	89 (85.6)	15 (14.4)
	Ampicillin	AMP	25	44 (31.4)	96 (68.6)	69 (66.3)	35 (33.7)
Tetracyclines	Tetracycline	T	30	115 (82.1)	25 (17.9)	96 (92.3)	8 (7.7)
Aminoglycosides	Streptomycin	S	300	109 (77.9)	31 (22.1)	70 (67.3)	34 (32.7)
	Kanamycin	K	30	91 (65)	49 (35)	32 (30.8)	72 (69.2)
Cephalosporins	Ceftriaxone	CRO	30	20 (14.3)	120 (85.7)	40 (38.5)	64 (61.5)
	Ceftazidime	CAZ	10	37 (26.4)	103 (73.6)	29 (27.9)	75 (72.1)
Carbapenems	Imipenem	IMI	10	0 (0)	140 (100)	43 (41.3)	61 (58.7)
	Ertapenem	ETP	10	2 (1.4)	138 (98.6)	19 (18.3)	85 (81.7)
Phenicols	Chloramphenicol	C	30	127 (90.7)	13 (9.3)	77 (74)	27 (26)
Foliate pathway inhibitor	Sulfamethoxazole/trimethoprim	TS	25	93 (66.4)	47 (33.6)	91 (87.5)	13 (12.5)

S: susceptible; R: resistance.

**Table 6:** Multiple antibiotic resistance patterns (MARPs) and MARI of *E*. *coli* isolates.

Pattern number	Number of antibiotics	MAR pattern	Meat sector		Total	MARI
			Formal	Informal		
1	3	A-TS-C	4	6	10	0.2
2	3	A-TS-AMP	11	2	13	0.2
3	3	A-AMP-CIP	7	4	11	0.2
4	3	TS-AMP-S	3	0	3	0.2
5	3	S-T-C	8	7	15	0.2
6	4	AMP-A-GM-TS	5	12	17	0.2
7	4	K-CAZ-S-AMP	6	1	7	0.2
8	4	CAZ-CTX-IMI-TS	0	9	9	0.2
9	5	ETP-C-IMI-AMP-CRO	5	5	10	0.3
10	5	TS-AMP-A-C-CAZ	0	3	3	0.3
11	6	AMP-IMI-CAZ-TS-T-C	1	2	3	0.3
12	6	K-AMP-S-A-T-IMI	2	0	2	0.3
13	6	IMI-NOR-S-T-A-TS	5	4	9	0.3
14	6	K-S-CRO-CAZ-AMP-TS	8	2	10	0.3
15	7	A-AMP-TS-K-IMI-NOR-C	0	8	8	0.4
16	7	K-S-IMI-C-T-A-NOR	8	4	12	0.4
17	8	ETP-C-IMI-T-A-AMP-CAZ-S	9	6	15	0.4
18	8	AMP-T-TS-C-K-CRO-ETP-A	0	2	2	0.4
19	8	CRO-ETP-A-S-IMI-K-AMP-T	0	1	1	0.4
20	9	T-CRO-K-AMP-NOR-TS-S-ETP-CAZ	5	3	8	0.5
21	9	CIP-AMP-T-TS-C-S-CAZ-IMI-A	0	8	8	0.5
22	10	C-IMI-TS-T-CRO-ETP-NOR-K-A-AMP	4	9	13	0.5
Total			91	98	189	

## Data Availability

All data that support the conclusions of this study are described in the article.
